# BananaLSD: A banana leaf images dataset for classification of banana leaf diseases using machine learning

**DOI:** 10.1016/j.dib.2023.109608

**Published:** 2023-09-22

**Authors:** Shifat E. Arman, Md. Abdullahil Baki Bhuiyan, Hasan Muhammad Abdullah, Shariful Islam, Tahsin Tanha Chowdhury, Md. Arban Hossain

**Affiliations:** a Department of Robotics and Mechatronics Engineering, University of Dhaka, Dhaka-1000, Bangladesh; b Department of Plant Pathology, Bangabandhu Sheikh Mujibur Rahman Agricultural University, Gazipur 1706, Bangladesh; c GIS and Remote Sensing Lab, Department of Agroforestry and Environment, Bangabandhu Sheikh Mujibur Rahman Agricultural University, Gazipur 1706, Bangladesh

**Keywords:** Banana leaf, Disease detection, Image classification, Machine learning, Deep learning, Computer vision, Plant pathology

## Abstract

Bananas, one of the most widely consumed fruits globally, are highly susceptible to various leaf spot diseases, leading to significant economic losses in banana production. In this article, we present the Banana Leaf Spot Diseases (BananaLSD) dataset, an extensive collection of images showcasing three prevalent diseases affecting banana leaves: Sigatoka, Cordana, and Pestalotiopsis. The dataset was used to develop the BananaSqueezeNet model [1]. The BananaLSD dataset contains 937 images of banana leaves collected from banana fields, which were then further augmented to generate another 1600 images. The images were acquired using three smartphone cameras in diverse real-world conditions. The dataset has potential for reuse in the development of machine learning models that can help farmers identify symptoms early. It can be useful for researchers working on leaf spot diseases and serve as motivation for further researches.

Specifications Table

**Table:** 

Subject	Computer Science, Data Science, Agricultural Sciences
Specific subject area	Computer Vision and Pattern Recognition; Plant Pathology; Agronomy and Crop Science; Applied Machine Learning; Disease Detection;Image Recognition
Type of data	Image (JPEG)
Data format	Raw and Processed
Data collection	The data for this study were gathered from two primary sources: the banana farm located at Bangabandhu Sheikh Mujibur Rahman Agricultural University and the neighboring fields of local farmers in Gazipur, Bangladesh. The images of the disease affected leaves were collected during the month of June 2021. These images were then labelled by an expert plant pathologist.
Data source location	Institution: Bangabandhu Sheikh Mujibur Rahman Agricultural University and adjacent banana fields City/Town/Region: Gazipur Country: Bangladesh Latitude and longitude : 24.036016 and 90.401085
Data accessibility	Repository Name: Mendeley Data Data Identification Number (DOI):10.17632/9tb7k297ff.1 Direct URL to the Dataset: https://data.mendeley.com/datasets/9tb7k297ff/1
Related research article	Md. A. B. Bhuiyan, H. M. Abdullah, S. E. Arman, S. Saminur Rahman, & K. Al Mahmud (2023). BananaSqueezeNet: A very fast, lightweight convolutional neural network for the diagnosis of three prominent banana leaf diseases. In Smart Agricultural Technology (Vol. 4, p. 100,214). Elsevier BV. https://doi.org/10.1016/j.atech.2023.100214

## 1 Value of the Data

• This dataset (BananaLSD) is collected in natural environment with variable light and atmospheric condition. This inconsistency makes the dataset more diverse.
• The dataset could be useful for plant pathologists as well as producers for detecting disease symptoms.
• The dataset could be useful for early leaf spot disease detection and appropriate intervention for banana growers in the context of smart farming.
• The dataset facilitates the low-cost banana leaf spot disease detection model.

## 2 Data Description

**Fig. 1:**
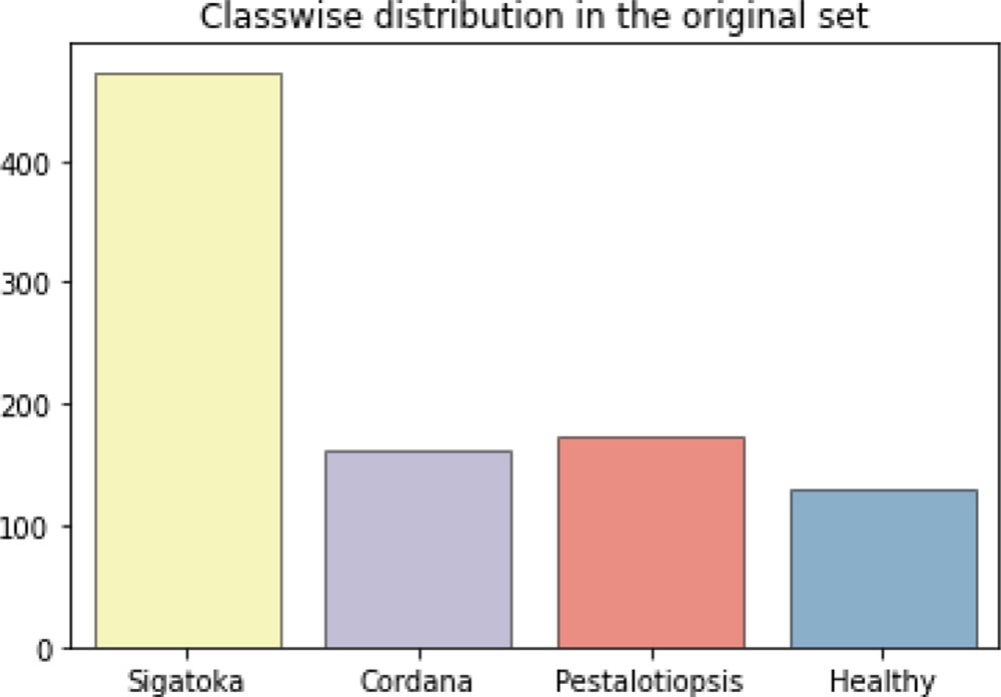
Distribution of the classes in the original set.

The BananaLSD dataset contains annotated images of three categories of diseased banana leaves: Pestalotiopsis, Sigatoka, and Cordana. They are accompanied by a set of images of healthy banana leaves. The four classes total to 937 images. The distribution of these images is illustrated in Fig. 1. Several augmentation techniques were then performed randomly on the original images to diversify the collection and negate the imbalance of class distribution. Each augmented class contains 400 images. The classwise distribution of both sets is presented in Table 1. The images were labelled by an expert plant pathologist. All images were taken manually with three smartphone cameras. The detailed information and characteristics of each disease are presented in Table 2.

**Table 1:** Classwise distribution of the dataset (Adapted from [1]).

Class Name	No. of Images	
	Raw Images	Augmented Images
Pestalotiopsis Leaf Blight	173	400
Sigatoka	473	400
Cordana Leaf Spot	162	400
Healthy	129	400
Total	937	1600

**Table 2:** Detailed description and random samples of each class.

Disease name	Description	Visualization
Sigatoka disease	Sigatoka is one of the most prominent fungal diseases that affects banana plants and is responsible for yield reduction of up to 50%. There are two types of Sigatoka: Black Sigatoka and Yellow Sigatoka. Black Sigatoka, caused by the fungus Mycosphaerella fijiensis, is more destructive and causes significant amount of banana yield loss. Yellow Sigatoka, cased by the fungus Mycosphaerella musicola, is comparatively less destructive [2]. Black Sigatoka mainly infects banana leaves. The symptom is characterized by the appearance of small, dark brown to black spots on the leaves. Leaves gradually turn yellow and eventually die as these spots enlarge and coalesce. The fungus of Black Sigatoka can spread rapidly in favourable environmental conditions. This disease was first reported in Fiji in 1912, and since then, it spread to other parts of the world. Black Sigatoka mainly prefers humid and rainy weather. This disease can also be spread by wind, water, and contaminated tools or equipment. Thus, the control of the disease is difficult.	
Cordana leaf spot	Cordana leaf spot is a prevalent fungal disease that affects the leaves of banana plants. It is caused by Cordana musae. The disease is characterized by the appearance of circular, water-soaked spots on the leaves, which eventually turn brown and necrotic. As the disease progresses, the spots may coalesce, leading to extensive leaf damage and defoliation, ultimately affecting plant growth and fruit yield. This disease is commonly spread in an area with high humidity and frequent rainfall. It can also spread through the infected planting materials [3].	
Pestalotiopsis leaf blight	Pestalotiopsis leaf blight is a common fungal disease that affects various plant species, including banana plants. It infects the plant's leaves, leading to brown, water-soaked lesions on the leaf surface. These lesions can expand and coalesce, resulting in extensive leaf damage and defoliation, negatively impacting plant growth and fruit yield. This disease commonly occurs in warm and humid environments. It can also spread through the planting of infected material [4,5].	

**Fig. 2:**
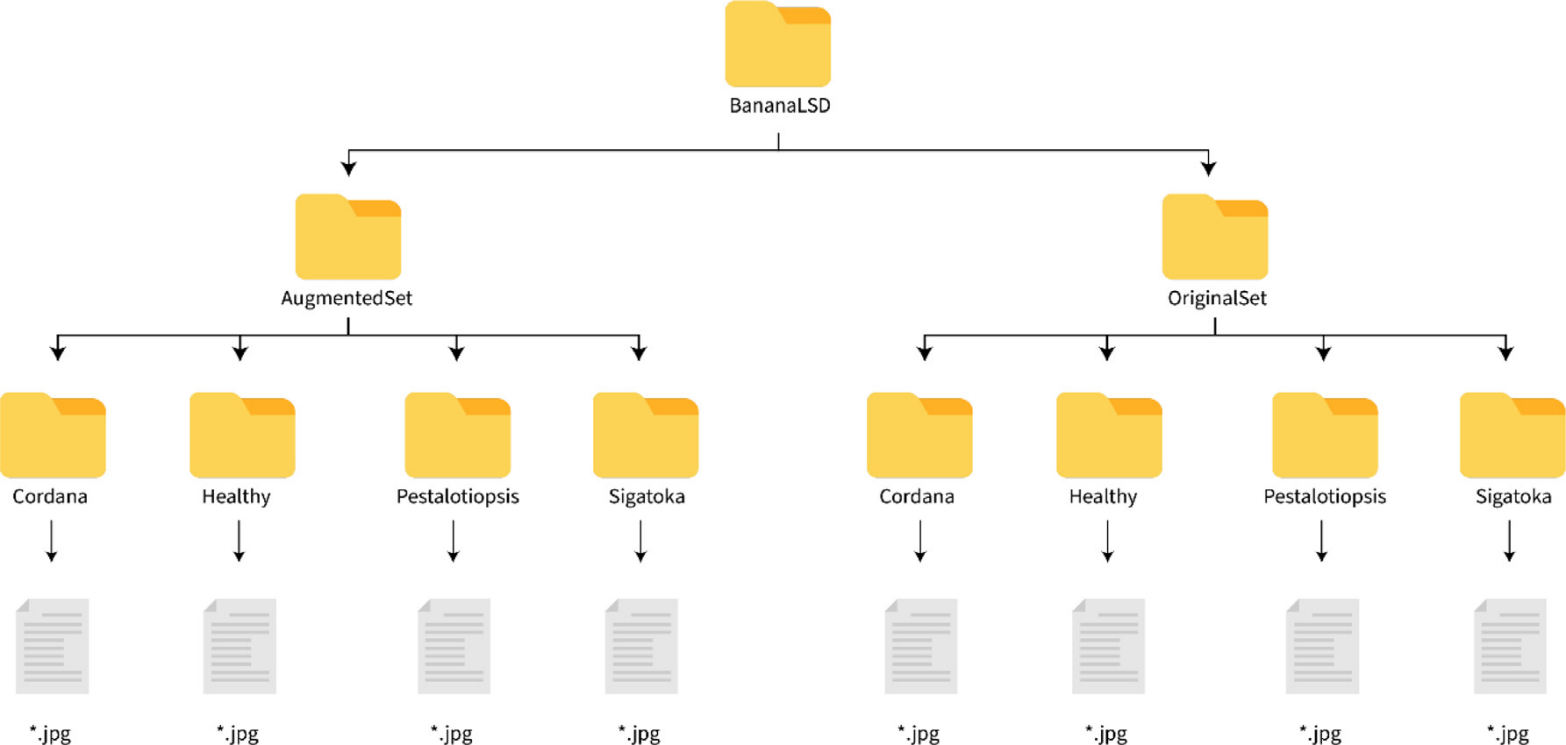
Directory structure of the dataset.

The dataset folder contains two folders, one for the original set and the other for augmented set. Both of these folders contain four subfolders - cordana, pestalotiopsis, sigatoka, and healthy - one for each of the classes. The directory structure is illustrated in Fig. 2.

## 3 Experimental Design, Materials and Methods

### 3.1 Data gathering

**Fig. 3:**
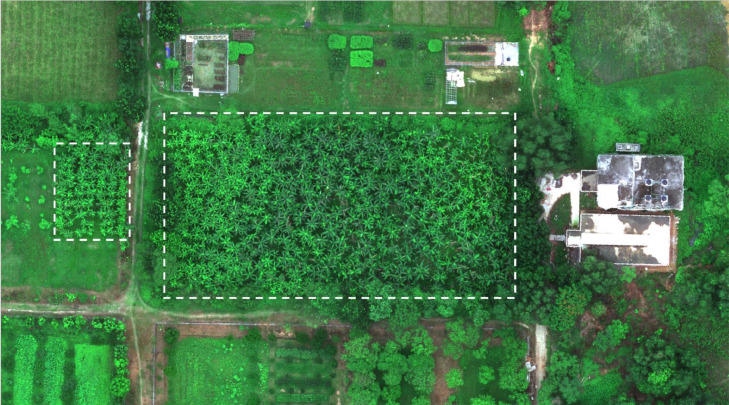
The banana field situated in Bangabandhu Sheikh Mujibur Rahman Agricultural University.

The data were gathered in natural light using high-resolution rear cameras of three smartphones, as shown in Table 3. The study area is shown in Fig 3. The images were purposefully captured with different or no backgrounds, varying lighting conditions, and different angles to create a diverse dataset. All images were then labelled by a plant pathologist. The data acquisition process took place during June 2021.

**Table 3:** Camera specification details.

Particulars	Camera 1	Camera 2	Camera 3
Camera maker	HUAWEI	Xiaomi	Xiaomi
Camera model	ELE-L29	Redmi 8A Dual	Redmi Note 9
Resolution	2736 × 3648	3120 × 4208	2250 × 4000
Color space	RGB	RGB	RGB
Focal length	5.58 mm	3.789 mm	4.7 mm
F-number	f/1.8	f/2.2	f/1.8
Exposure time	1/532 s	1/100 s	1/100 s

### 3.2 Data preprocessing

All images in the raw set were resized to 224 × 224 pixels, a standard resolution for building image classification models. We then performed augmentation on the resized images. Image augmentation is used to expand a dataset artificially to account for limited data [6]. Deep learning models tend to overfit toward classes with higher samples. A model trained with images captured from only one perspective will tend to perform worse on another image of the same object taken from a different angle. Augmentation ensures diversity in the dataset and addresses data imbalances that are present. The following augmentation techniques were applied randomly within the raw dataset.

(1) **Crop:** We applied random crops on the images. As our data contained only one label per image, the crops were deemed to be a label-preserving transformation. Cropping generally reduces the size of the input. In our scenario, it additionally fills the dataset with images of varying height and width dimensions by extracting a central patch from each image.
(2) **Horizontal flip:** Flipping is usually not considered a label-preserving transformation on datasets involving text recognition. But our scenario is unaffected by the flipping, hence it is considered safe to implement. Implementing this augmentation is straightforward and has demonstrated its effectiveness with datasets such as ImageNet [7].
(3) **Translation:** Shifting images is a useful technique to mitigate positional bias in the dataset. This technique provides similar effects to cropping but cropping reduces the size of the input whereas translations preserve spatial dimensions [7].
(4) **Shear:** Shear distorts the image along a certain axis. This is done to mostly create or rectify perception angles.
(5) **Rotate shear:** This is similar to shear. But it also applies a rotation to the image. Again, as our use case is not affected by the orientation of the sample, this is considered label-preserving.
(6) **Linear contrast adjustment:** This is the only color space transformation done on the dataset. Lighting biases are one of many challenges that occur in image identification tasks. LCA is applied to fix overly bright or dark samples.
(7) **Gaussian blurring:** Blurring images can make the deep learning model more forgiving towards images riddled with motion blur during testing.

400 augmented images were generated for each class. The raw set was then split into training, test, and validation sets. The augmented set was used only for training.

## Limitations

Inclusion of more classes and samples from countries other than Bangladesh can improve the diversity of the dataset.

## Ethics Statements

This article does not contain any studies involving animals or human beings. No data collected from social media platforms were used.

## CRediT authorship contribution statement

**Shifat E. Arman:** Investigation, Methodology, Writing – original draft, Supervision, Writing – review & editing. **Md. Abdullahil Baki Bhuiyan:** Conceptualization, Supervision, Writing – review & editing. **Hasan Muhammad Abdullah:** Supervision, Conceptualization, Resources, Writing – review & editing. **Shariful Islam:** Data curation, Resources, Writing – original draft. **Tahsin Tanha Chowdhury:** Data curation, Resources, Writing – original draft. **Md. Arban Hossain:** Writing – original draft, Visualization, Writing – review & editing.

## Data Availability

Banana Leaf Spot Diseases (BananaLSD) Dataset for Classification of Banana Leaf Diseases Using Machine Learning (Original data) (Mendeley Data).

## References

[1] Bhuiyan Md.A.B., Abdullah H.M., Arman S.E., Saminur Rahman S., Al Mahmud K. (2023). BananaSqueezeNet: a very fast, lightweight convolutional neural network for the diagnosis of three prominent banana leaf diseases. Smart Agric. Technol..

[2] Surridge A.K.J., Viljoen A., Crous P.W., Wehner F.C. (2003). Identification of the pathogen associated with Sigatoka disease of banana in South Africa. Austral. Plant Pathol..

[3] Hernandez Restrepo M., GROENEWALD J.Z., CROUS P.W. (2015). Neocordana gen. nov., the causal organism of Cordana leaf spot on banana. Phytotaxa.

[4] Maharachchikumbura S.S.N., Guo L.-D., Chukeatirote E., Bahkali A.H., Hyde K.D. (2011). Pestalotiopsis—morphology, phylogeny, biochemistry and diversity. Fungal Divers..

[5] Bhuiyan Md.A.B., Islam S.M.N., Bukhari Md.A.I., Kader Md.A., Chowdhury Md.Z.H., Alam M.Z., Abdullah H.M., Jenny F. (2022). First report of pestalotiopsis microspora causing leaf blight of banana in Bangladesh. Plant Dis..

[6] Perez L., Wang J. (2017).

[7] Shorten C., Khoshgoftaar T.M. (2019). A survey on image data augmentation for deep learning. J. Big Data.

